# Oral and Stool Microbiome Coalescence and Its Association With Antibiotic Exposure in Acute Leukemia Patients

**DOI:** 10.3389/fcimb.2022.848580

**Published:** 2022-03-31

**Authors:** Samantha Franklin, Samuel L. Aitken, Yushi Shi, Pranoti V. Sahasrabhojane, Sarah Robinson, Christine B. Peterson, Naval Daver, Nadim A. Ajami, Dimitrios P. Kontoyiannis, Samuel A. Shelburne, Jessica Galloway-Peña

**Affiliations:** ^1^ Department of Veterinary Pathobiology, Texas A&M University, College Station, TX, United States; ^2^ Interdisciplinary Graduate Program in Genetics and Genomics, Texas A&M University, College Station, TX, United States; ^3^ Department of Pharmacy, Michigan Medicine, Ann Arbor, MI, United States; ^4^ Department of Statistics and Center for Biomedical Informatics, University of Missouri, Columbia, MO, United States; ^5^ Department of Infectious Disease, Infection Control and Employee Health, The University of Texas MD Anderson Cancer Center, Houston, TX, United States; ^6^ Department of Statistics, Rice University, Houston, TX, United States; ^7^ Department of Biostatistics, The University of Texas MD Anderson Cancer Center, Houston, TX, United States; ^8^ Department of Leukemia, The University of Texas MD Anderson Cancer Center, Houston, TX, United States; ^9^ Department of Genomic Medicine, The University of Texas MD Anderson Cancer Center, Houston, TX, United States

**Keywords:** microbiome, coalescence, leukemia, oralization, antimicrobials

## Abstract

Failure to maintain segregation of oral and gut microbial communities has been linked to several diseases. We sought to characterize oral-fecal microbiome community coalescence, ectopic extension of oral bacteria, clinical variables contributing to this phenomenon, and associated infectious consequences by analyzing the 16S rRNA V4 sequences of longitudinal fecal (n=551) and oral (n=737) samples from 97 patients with acute myeloid leukemia (AML) receiving induction chemotherapy (IC). Clustering observed in permutation based multivariate analysis of variance (PERMANOVA) of Bray-Curtis dissimilarity and PCoA plot of UniFrac distances between intra-patient longitudinal oral-stool sample pairs suggested potential oral-stool microbial community coalescence. Bray-Curtis dissimilarities and UniFrac distances were used to create an objective definition of microbial community coalescence. We determined that only 23 of the 92 patients exhibited oral-stool community coalescence. This was validated through a linear mixed model which determined that patients who experienced coalescence had an increased proportion of shared to unique OTUs between their oral-stool sample pairs over time compared to non-coalesced patients. Evaluation of longitudinal microbial characteristics revealed that patients who experienced coalescence had increased stool abundance of *Streptococcus* and *Stenotrophomonas* compared to non-coalesced patients. When treated as a time-varying covariate, each additional day of linezolid (HR 1.15, 95% CI 1.06 – 1.24, P <0.001), meropenem (HR 1.13, 95% CI 1.05 – 1.21, P = 0.001), metronidazole (HR 1.13, 95% CI 1.05 – 1.21, P = 0.001), and cefepime (HR 1.10, 95% CI 1.01 – 1.18, P = 0.021) increased the hazard of oral-stool microbial community coalescence. Levofloxacin receipt was associated with a lower risk of microbiome community coalescence (HR 0.75, 95% CI 0.61 – 0.93, P = 0.009). By the time of neutrophil recovery, the relative abundance of Bacteroidia (P<0.001), Fusobacteria (P=0.012), and Clostridia (P=0.013) in the stool were significantly lower in patients with oral-gut community coalescence. Exhibiting oral-stool community coalescence was associated with the occurrence of infections prior to neutrophil recovery (P=0.002), as well as infections during the 90 days post neutrophil recovery (P=0.027). This work elucidates specific antimicrobial effects on microbial ecology and furthers the understanding of oral/intestinal microbial biogeography and its implications for adverse clinical outcomes.

## Introduction

Despite the mouth and intestine being linked by saliva and ingested food, the oral cavity and gut harbor distinct microbial communities (Human Microbiome Project, 2012; Segata et al., 2012; Ding and Schloss, 2014; Franzosa et al., 2014; Rashidi et al., 2021). Segregation of the oral and gut microenvironments are thought to be maintained by gastric and bile acids, such that low numbers of viable oral bacteria reach the gut (Martinsen et al., 2005; Tennant et al., 2008; Ridlon et al., 2014; Park et al., 2021). Failure to maintain this oral-gut barrier has been linked to several diseases including inflammatory bowel disease (IBD), liver cirrhosis, colon cancer, and rheumatoid arthritis (Qin et al., 2014; Zhang et al., 2015; Atarashi et al., 2017; Flemer et al., 2018; Huh and Roh, 2020).

Although the oral and gut microbiomes of an individual typically maintain unique compositions, there are several bacterial species that can colonize both the human mouth and intestines (Segata et al., 2012; Seedorf et al., 2014; Schmidt et al., 2019). Recently, the novel theory of the process of microbial “community coalescence” has been proposed, which is defined as a new microbial community arising from the admixture of two or more separate communities (Rillig et al., 2015; Lechon-Alonso et al., 2021). Although this terminology has been primarily applied to describe ecological community interchange in an environmental context (i.e. the mixing of population in aquatic environments, or rhizosphere communities), there is recent evidence that community coalescence can also occur within the human body with important consequences (Rillig et al., 2015). For example, several studies have shown that finding typical gut microbes at other body sites, such as lung or skin, is associated with pathologic states including acute respiratory distress syndrome and nosocomial infections (Dickson et al., 2016; Rogers et al., 2016). Additionally, high levels of oral bacteria have been found in stool samples from patients with Crohn’s disease, colorectal cancer, and rheumatoid arthritis (Qin et al., 2014; Zhang et al., 2015; Atarashi et al., 2017; Flemer et al., 2018). Moreover, it was recently reported that oralization of the gut microbiome during proton-pump inhibitor therapy was linked to intestinal inflammation, gut barrier dysfunction, and liver disease severity (Horvath et al., 2019; Horvath et al., 2021). Altogether, these findings suggest that proliferation of oral bacteria in the gut and the resulting microbiome community coalescence may be an important contributor to a range of human diseases and have implications for the structure and function of human associated microbial communities (Olsen and Yamazaki, 2019; Park et al., 2021).

Although a few microbiome studies have investigated non-contiguous body sites, the majority of such studies were performed in hospitalized patients and have focused on the contributions of the stool microbiome on clinical outcomes rather than the interaction between body sites (McDonald et al., 2016; Akrami and Sweeney, 2018). Consequently, there is limited knowledge about the relationship between oral and stool microbial communities in patients with hematologic cancer. Given that we had collected data on a sizeable cohort of such patients with simultaneous oral and stool longitudinal microbiome samples (Galloway-Pena et al., 2020), we sought to use this dataset to systematically define oral-fecal community coalescence, ectopic extension of oral bacteria, the microbiome and clinical variables contributing to these phenomena and the clinical consequences. Our underlying hypothesis was that gut decontamination *via* broad-spectrum antibiotics allows for community coalescence and oralization of the intestinal microenvironment resulting in increased risk of negative clinical outcomes.

## Materials and Methods

### Study Design, Participants, and Clinical Definitions

Longitudinal fecal (n=551) and oral (n=737) samples and respective 16S V4 rRNA sequences were derived from 97 adult patients with acute myeloid leukemia undergoing induction chemotherapy (i.e. initial treatment) at MD Anderson Cancer Center (MDACC) in Houston, TX from September 2013 to August 2015. Aspects of this cohort were previously published and 16S rRNA sequences deposited in the NCBI Sequence Read Archive (http://www.ncbi.nlm.nih.gov/sra) under the BioProject IDs PRJNA352060 and PRJNA526551 (Galloway-Pena et al., 2016; Galloway-Pena et al., 2017; Galloway-Pena et al., 2020). The study protocol was approved by the MDACC Institutional Review Board (PA13‐0339) and was conducted in compliance with the Declaration of Helsinki. Written informed consent was obtained from all participants before enrollment. Specimens were collected prior to chemotherapy initiation and twice weekly until neutrophil recovery (PMNs >500 cells/µl) as described previously (Galloway-Pena et al., 2016; Galloway-Pena et al., 2017; Galloway-Pena et al., 2020). Infections prior to neutrophil recovery were considered as microbiologically documented infections (MDI) or clinically documented infections (CDI) using established definitions as previously described (Galloway-Pena et al., 2016; Galloway-Pena et al., 2017). Infections post neutrophil recovery were considered as microbiologically documented infections (MDI) occurring within 90 days.

### Microbiome Analyses

16S rRNA V4 sequences were assigned to operational taxonomic units (OTUs) at 97% sequence similarity using the UPARSE pipeline and aligned to the SILVA SSURef_NR99_119 database with calculations of α- and β-diversity metrics of microbiome communities conducted in R using the Phyloseq package as previously described (McMurdie and Holmes, 2013; Quast et al., 2013). Differences in community structure were visualized using principal coordinate analysis (PCoA) of the Bray-Curtis dissimilarities, using 95% confidence ellipses of the oral samples and fecal samples separately with the P–value and coefficient of determination obtained by permutation based multivariate analysis of variance (PERMANOVA) (Anderson, 2001). PCoA plots of the weighted and unweighted UniFrac distances were generated in R using the ‘ggforitfy’ package (https://cran.r-project.org/web/packages/ggfortify/index.html). Boxplots, heatmaps, hierarchical clustering, and statistical analyses were generated in R or the user interface ATIMA (Agile Toolkit for Incisive Microbial Analyses) at https://atima.research.bcm.edu/. Linear mixed models were constructed to assess trends in oral and stool Shannon diversity over time and as a function of coalescence using the function lme from the nlme R package (https://cran.r-project.org/web/packages/nlme/index.html). For both models, the response variable was sample Shannon diversity, with fixed effects for the number of days on chemotherapy, coalescence status, and their interaction. A mixed linear effect model was used to compare the proportion of shared OTUs to unique OTUs over time and as a function of coalescence status. The random effect was the patients, the fixed effects were the days on chemotherapy, coalescence status, and the interaction between the two. R was used to plot the model estimates using the lme4 R package (https://cran.r-project.org/web/packages/lme4/index.html). Differential enrichment of bacterial taxa was estimated using a pairwise Mann-Whitney test whereas differences between timepoints were assessed using Wilcoxon matched-pairs signed rank test. Adjustment for multiple hypothesis testing was done within each taxonomy level by adjusting for false discovery rate (FDR) using Benjamini-Hochberg (BH) method (Benjamini and Hochberg, 1995). Mixed models for repeated measures were used to test if relative abundances of typical oral taxa were significantly different between the oral and stool of coalesced and non-coalesced patients, where the fixed effects were sampling location (oral vs stool) and time, and the random effects was the patient. Graphs of weighted UniFrac distance and relative abundance comparisons were generated in GraphPad Prism 7. Intergroup differences at all taxonomic levels were analyzed by the linear discriminant analysis of effect size (LEfSe) method (Segata et al., 2011) with default settings on the website https://huttenhower.sph.harvard.edu/galaxy/root.

### Antibiotic Use Assessment, Definitions, and Statistical Analysis

Antibiotic receipt was extracted from a database maintained by the Division of Pharmacy at MD Anderson Cancer Center. AML patients receiving IC were routinely prescribed a prophylactic fluoroquinolone or cephalosporin prior to the initiation of therapy. In this study, 100% of patients received routine prophylaxis, with the majority of baseline stool and oral samples taken after the patient had already started prophylactics. However, patients were excluded from the study if they had an active infection or were being treated with broad-spectrum antibiotics at time of enrollment.

In order to minimize the multiplicity of testing induced by including rarely used antibiotics, as well as to avoid overcompensating for those antibiotics that were administered more than once a day, we constrained the antibiotic administration data for all analyses by day and prevalence. An antimicrobial therapy day was defined as any single calendar day on which an antibiotic was administered, regardless of dose, route, or frequency. Antibiotic use was assessed at the individual drug level and considered as both any use (i.e., one or more days of therapy) and cumulative use (i.e., total days of therapy during the study period). Patients were assessed for antibiotic use from start of chemotherapy until neutrophil recovery or until the time of microbiome coalescence, as defined below. Only antibiotics given to >15% of the cohort were analyzed to allow for reliable effect estimates and sufficient statistical power.

To account for the time-varying nature of antibiotic use, a time-varying Cox proportional hazards model was used, with patients censored at neutrophil recovery or death. The time-varying Cox proportional hazards model accounts for immortal time bias and allows for an assessment of risk of coalescence associated with each additional day of antibiotic exposure (Stevens et al., 2011; Munoz-Price et al., 2016). Pearson’s χ^2^ test was used to determine whether there was a significant difference between the frequency of infection between those who did and did not exhibit oral-stool coalescence. Antibiotic and infection related statistical analyses were performed using Stata v13.1 (StataCorp LP, College Station, TX).

## Results

### Observation of Potential Oral-Stool Microbial Community Coalescence

**Figure 1:**
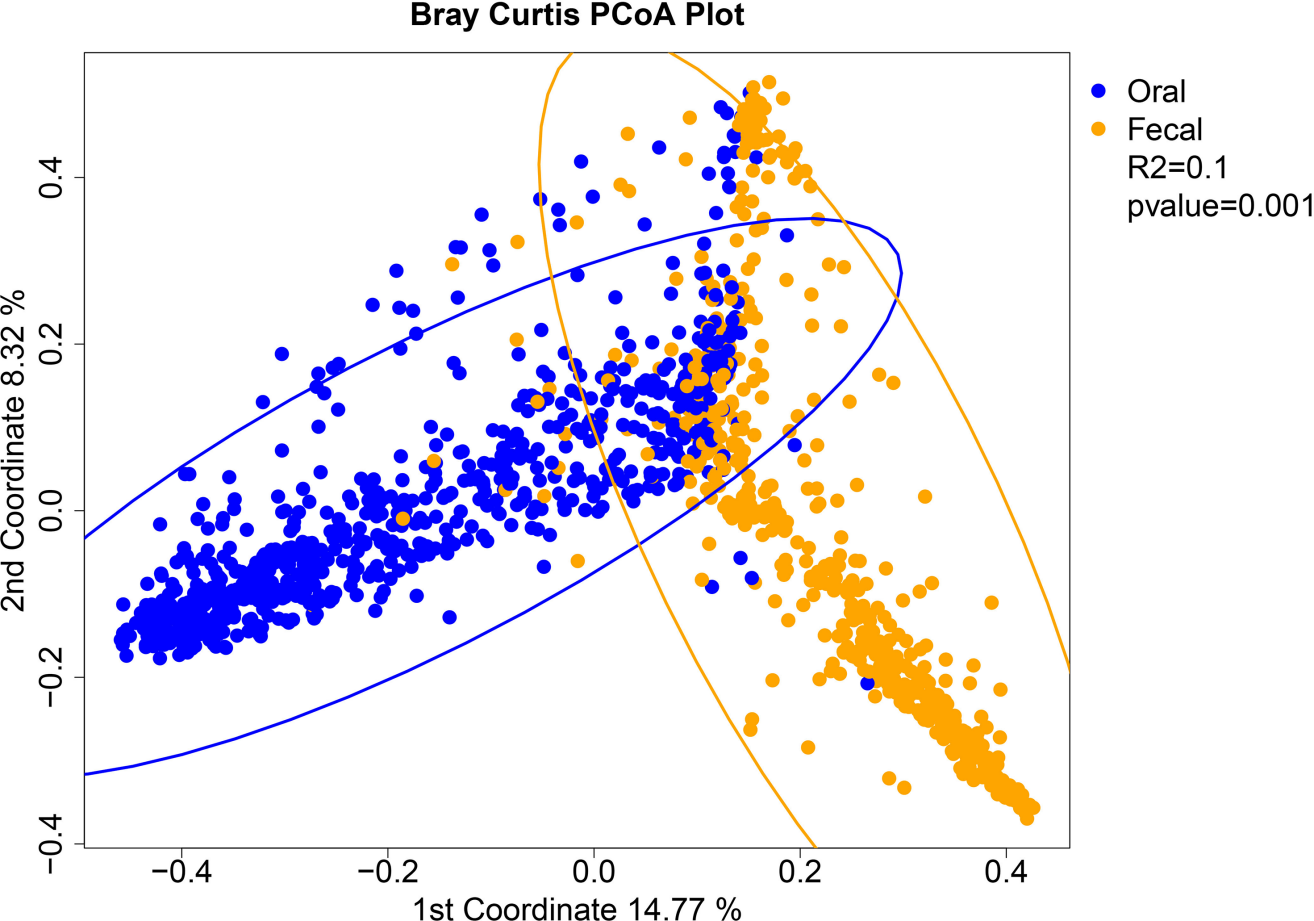
Analysis of beta-diversity demonstrates coalescence between oral and stool communities among leukemia patients undergoing induction chemotherapy. Principal coordinates analysis plot of the Bray-Curtis dissimilarity with 95% confidence ellipses of the 737 oral samples and 551 fecal samples separately. Oral (blue) and stool (orange) samples are colored by sample site. The P-value and coefficient of determination are derived from permutation based multivariate analysis of variance (PERMANOVA) using the Bray-Curtis dissimilarity matrix for all samples.

**Figure 2:**
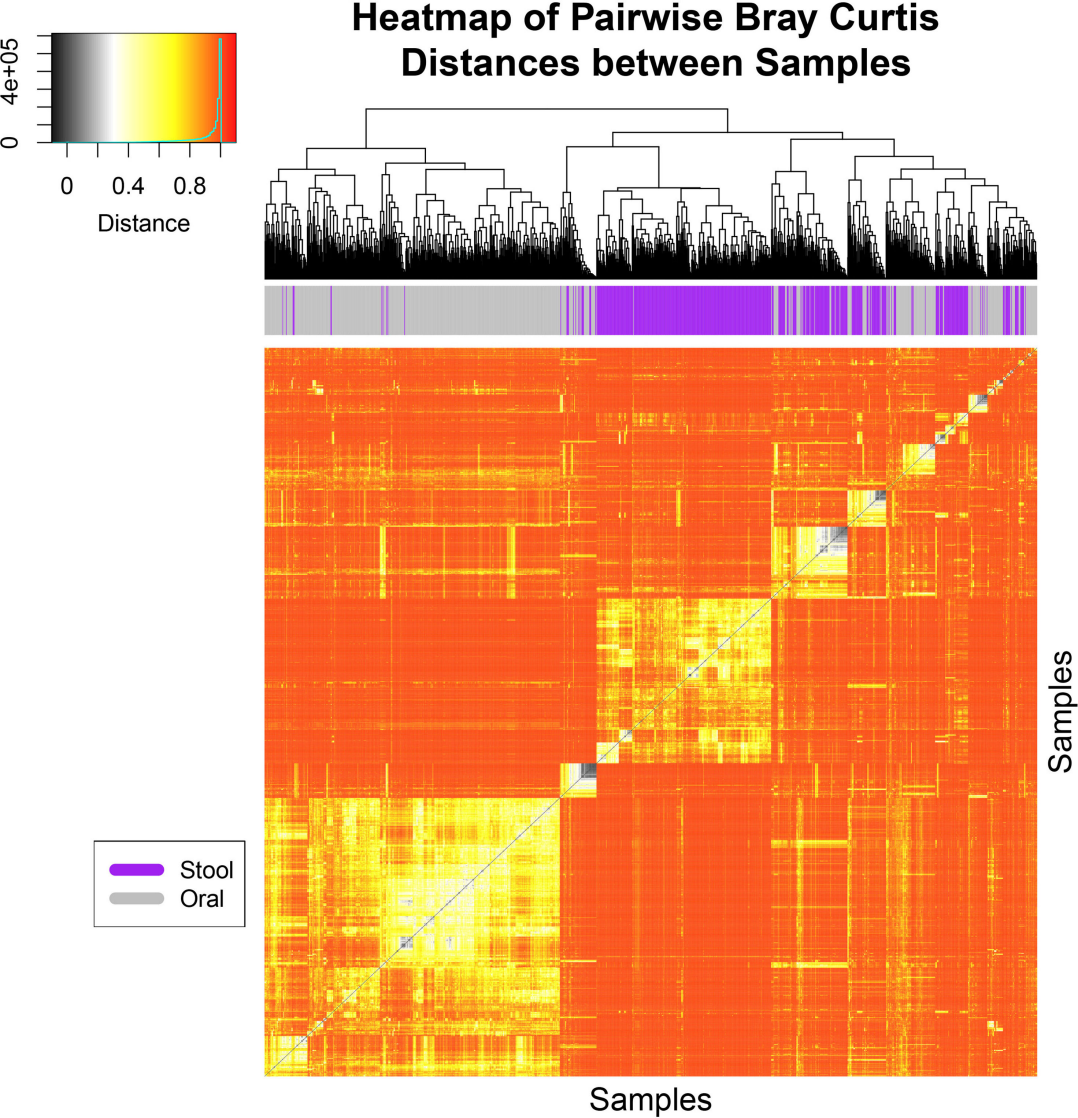
Hierarchical clustering analysis of Bray-Curtis distances shows three major clusters. A heat-map and hierarchical clustering dendrogram is shown based on pairwise Bray-Curtis distances for all samples collected from patients and colored at the top by body site. Hierarchical clustering was conducted for both axes, and is only visualized on the x-axis. Three major clusters of samples can be seen where the oral samples (left in gray), stool samples (middle in purple) clustered separately from a third group which had a mixture of stool and oral samples (right in both gray and purple). The heat-map is colored from black (0) to red (1) for the Bray-Curtis Distances in which specific values and counts are seen in the inlaid legend.

To determine whether oral-stool microbiome coalescence was occurring in our cohort, we first constructed a principal coordinates (PCoA) plot using different β-diversity metrics to show the variation among all 1,288 samples. This analysis demonstrated that there was primary clustering by body site, with the majority of samples segregating by the oral (blue) and stool (orange) habitats as has been previously observed (**Figures 1**, **S1**, **S2**) (Human Microbiome Project, 2012). Permutation based multivariate analysis of variance (PERMANOVA) using Bray-Curtis dissimilarity showed a significant difference in microbiome composition between the two sample types (P< 0.001) with a coefficient of determination equal to 0.1 However, ~25% of the oral and stool samples coexisted in the two-dimensional space of the principal coordinates plot (i.e. were present in both “oral” and “stool” ellipses concurrently, **Figure 1**). This was also observed in the PCoA plot of unweighted and weighted UniFrac distances (**Figures S1** and **S2**). Three distinct clusters were identified through hierarchical clustering of samples followed by inspection of the resulting heatmap of pairwise Bray Curtis distances between samples. The oral samples (left) and stool samples (middle) clustered separately from a third group (right) which had a mixture of stool and oral samples (**Figure 2**). For 39 of patients there was both an oral and stool sample in the “mixture” group indicating that such patients were potentially demonstrating community coalescence *via* a mixing of body site communities over the course of induction chemotherapy.

### Defining Oral and Stool Community Coalescence Within the Cohort

In order to ascertain which patients had inter-body site community merging during their course of chemotherapy, we next sought to determine an objective definition of microbial community coalescence between oral and stool sites. First, we determined the UniFrac distance for each oral-stool sample combination within each individual patient (i.e. the complete distance matrix for all longitudinal stool and oral samples from the same patient). We then plotted the minimum UniFrac distance between any longitudinally collected stool and oral sample pairs within a single patient (**Figure S3**). As five patients did not have sufficient longitudinal stool sampling to perform this analysis (greater than two pairs), 92 patients were included in the analysis.

Given that the minimum UniFrac distances were not normally distributed across subjects (Shapiro Wilk’s test P <0.001) we summarized the minimum UniFrac distances using median and interquartile ranges. The median of the minimum UniFrac distance was 0.38 with an interquartile range of 0.26 to 0.44. Due to the distribution of the data within our cohort, which gave no clear distinction or outliers, we defined a patient exhibiting microbial community coalescence between oral and stool sites as any patient with a minimum distance in the bottom quartile (**Figure S3**). Using this definition, we classified 23 patients as displaying microbial community coalescence between oral and stool sites. Among patients that met this definition, the range of the minimum UniFrac distance between any longitudinally collected stool and oral sample pair within a patient was 0.005-0.257, with a median of 0.181. Among patients who did not coalesce, the range was 0.272-0.594, with a median of 0.41. The median time to the highest degree of coalescence in these patients, as determined by minimum UniFrac distance between any intra-patient oral-stool pair, was 22 days from chemotherapy initiation. We sought to validate our definition by determining of how many of these 23 patients had samples that were in the mixture ellipse identified in **Figure 1**. Consistent with these patients having oral-stool coalescence, 20 of the 23 patients identified as coalescing contributed samples contained in the overlap between the two ellipses, while only 21 of the 66 identified as non-coalescing patients had samples placed within the overlap of the ellipses

**Figure 3:**
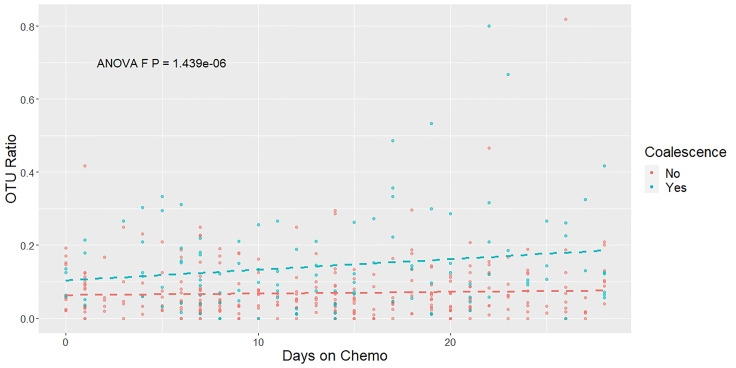
The ratio of shared to unique OTUs between oral and stool pairs increases over time. A mixed linear effect model was constructed to compare the OTUs present in each patient’s stool and oral samples longitudinally throughout chemotherapy treatment. The ratio of OTUs was calculated by comparing the number of OTUs that were present in both the oral and stool sample in a particular time point compared with the number of OTUs that were unique at that same time point. Colors indicate the patients’ coalescence status: blue for coalesced, red for not coalesced. Dashed lines show the estimated linear trend from the mixed linear effect model. The P-value is derived from an ANOVA test.

To further validate our categorization of the patients as coalesced or not coalesced based on using the minimum UniFrac distance between oral and stool pairs, we analyzed the oral and stool samples in the context of shared and unique OTUs between paired samples over time. If the oral and stool microbial communities were coalescing over time, we would expect that intra-patient oral and stool sample pairs would share increasingly more of the same OTUs over the average course of treatment (0-28 days), compared to sample pairs for patients who were not experiencing coalescence. Therefore, we plotted the ratio of shared to unique OTUs between oral and stool pairs coalesced (blue) and non-coalesced (red) patients over 28 days (**Figure 3**). We then fit a linear mixed model and confirmed that patients who were classified as experiencing coalescence have an increased proportion of shared to unique OTUs between their oral and stool sample pairs over time compared to non-coalesced patients (P<0.001) (**Figure 3**).

### Characterizing Microbial Community Changes of Patients Who Exhibited Oral and Stool Community Coalescence

Coalescence can result in symmetrical outcomes, with equal contribution of the two communities, or asymmetrical, in which there is a dominance of one community over the other (Gilpin, 1994; Sierocinski et al., 2017; Castledine et al., 2020). Interestingly, early mathematical models showed when two communities merge after barrier removal, asymmetrical dominance is likely to occur (Gilpin, 1994). Thus, we sought to distinguish between these possibilities by analyzing the diversity of longitudinally coalesced samples. Consistent with the second scenario, at the time of the highest degree of coalescence both oral and stool α-diversity were low. Specifically, for oral samples the median observed OTUs and Shannon diversity were significantly lower at the time of maximal coalescence relative to those measures in the remaining overall entire cohort (OTUs 19 vs. 29 and Shannon diversity 1.07 vs. 1.81, P < 0.001 for both using a Mann-Whitney test). Similarly, for stool samples, the median observed OTUs and Shannon diversity were also significantly lower at time of highest coalescence compared to the overall cohort (OTUs 14 vs. 24, P=0.01, and Shannon diversity 0.96 vs. 1.77, P < 0.001 using a Mann-Whitney test). Additionally, only one or two organisms were typically present in high abundance in the oral and the stool for coalesced samples. Of the 23 patients who experienced coalescence, 87% experienced at least one domination event (>30% relative abundance of reads attributed to one genus) of the same genera in both their oral and stool. The most common genera contributing to domination at both sites in patients with coalescence were *Streptococcus* (52%) followed by *Staphylococcus* (17%) (**Supplementary Table 1**).

**Figure 4:**
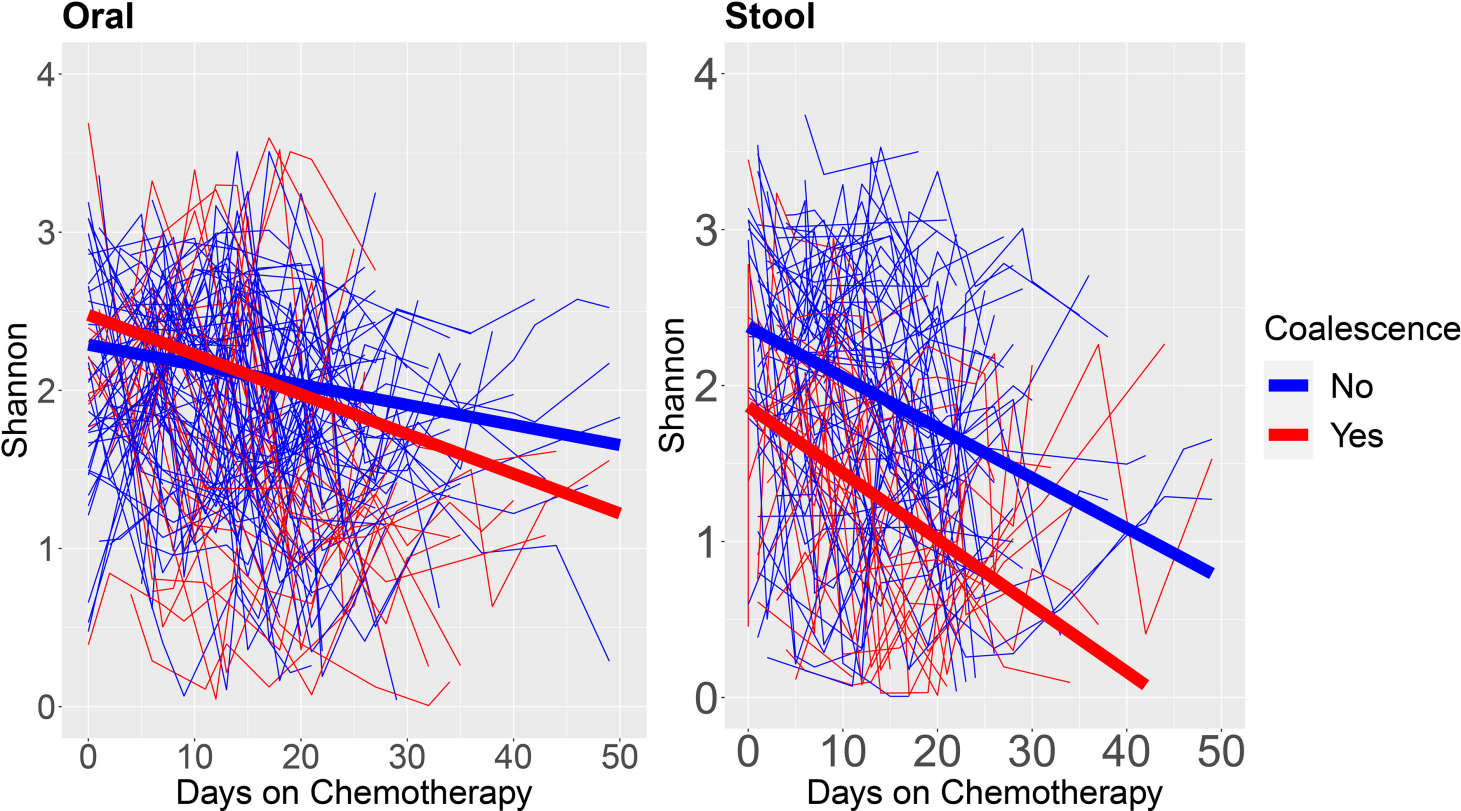
α-diversity over time stratified by patients who do and do not experience oral-stool microbial community coalescence. We fit linear mixed models on the oral and stool Shannon Diversity by days on chemotherapy to neutrophil recovery for each individual patient. Blue lines are from patients who do not coalesce, whereas red lines are from patients who coalesce. The thick blue line shows the estimated linear trend from the linear mixed model for patients who do not coalesce, and the thick red line shows the estimated linear trend from the linear mixed model for patients who coalesce.

To further evaluate the longitudinal microbiome characteristics that were associated with coalescence, we fit linear mixed models on Shannon diversity over time as a function of coalescence for oral and stool samples (**Figure 4**). In the patients that coalesced, the oral sample α-diversity has a steeper decrease over time (coefficient for interaction of coalescence and time = -0.017, p = 0.0015). For stool, although the slopes were similar (i.e., the interaction of coalescence and time was not significant) the main effect of coalescence was significant (coefficient: -0.476, p = 0.0172), where the stool samples of patients that coalesced had an overall lower α-diversity over time, which appeared to be primarily driven by the patients who eventually coalesced having an initially lower α-diversity in their stool samples. Race, age, gender, and administered antineoplastics and chemotherapies were tested for their association with changes in Shannon Diversity as well as coalescence status, however none were found to be significantly associated with bacterial community coalescence in univariate statistical modeling, suggesting they are not likely potential confounders.

**Figure 5:**
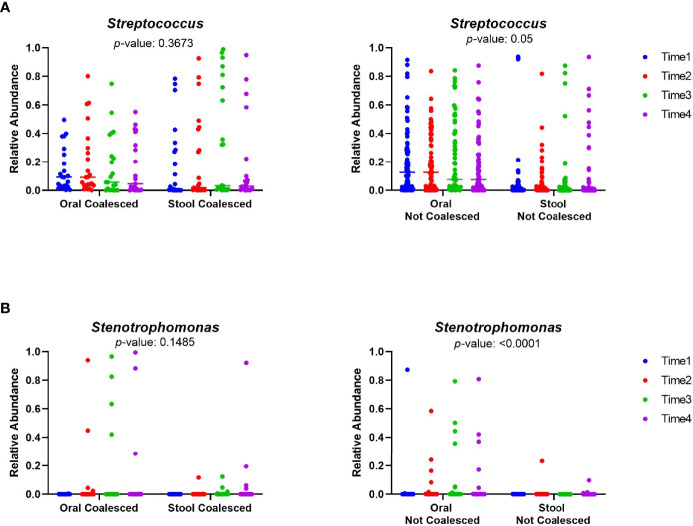
The relative abundances of *Streptococcus* and *Stenotrophomonas* are not significantly different between the oral and stool of coalesced patients. The relative abundance of selected oral bacteria **(A)** *Streptococcus* and **(B)** *Stenotrophomonas* was plotted for oral and stool samples. The relative abundance within each sampling time points for both oral and stool is plotted individually. The color scale indicates sampling time point for each of the patient samples, where blue is the first timepoint sampled, and purple is the last sampling time point. P-values were calculated between oral and stool samples utilizing a mixed model for repeated measures.

Given that increased amounts of oral bacteria have been reported in the intestine in a number of diseases (Qin et al., 2014; Zhang et al., 2015; Atarashi et al., 2017; Flemer et al., 2018; Huh and Roh, 2020), we next sought to determine if coalesced patients had greater abundance of different oral taxa in their stool than non-coalesced patients. Using longitudinal sample timepoints 1-4 for all patients (those were present for all) we tested for particular oral bacteria previously associated with oralization of the gut in other disease phenotypes such as *Streptococcus*, *Veillonella*, *Oribacterium*, *Neiseeria*, *Stenotrophomonas*, *Actinomyces*, *Leptotrichia*, and *Fusobacterium*. As expected, *Veillonella*, *Oribacterium*, *Neisseria*, *Actinomyces*, *Leptotrichia*, and *Fusobacterium* abundances were significantly higher in oral samples than stool in both coalesced and non-coalesced patients (**Figure S4**) (Segata et al., 2012; Horvath et al., 2019; Rashidi et al., 2021; Park et al., 2021). However, *Streptococcus* (P=0.367) and *Stenotrophomonas* (P=0.149) had no significant difference between oral and stool sample abundance in coalesced patients (**Figure 5**), indicating their abundance in the stool in coalesced patients is abnormally high.

### Determining Baseline Microbiome Features Associated With Developing Coalescence

**Figure 6:**
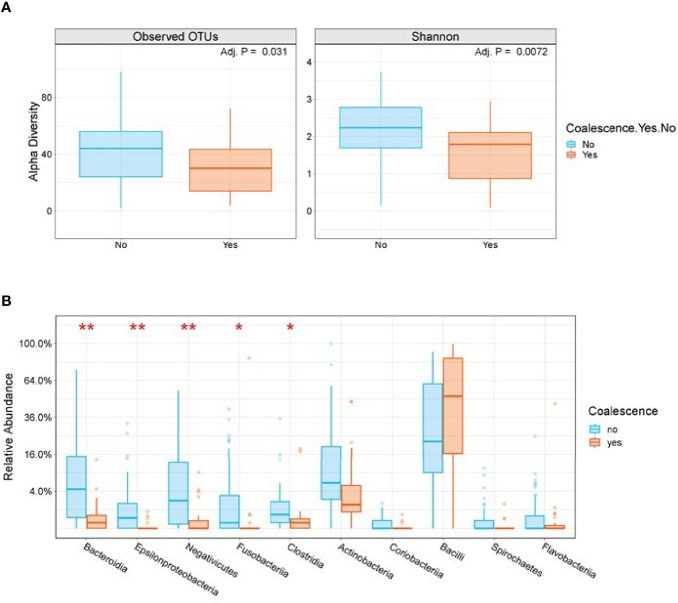
Stool α-diversity and taxonomic differences are seen among patients who do and do not exhibit oral-stool community coalescence. **(A)** A box plot of baseline stool Observed OTUs and Shannon Diversity segregated by those who do and do not go on to exhibit oral-stool microbial community coalescence. P-value is based on Mann-Whitney test with FDR adjustment using BH method. **(B)** Box plots of end of study stool samples segregated by those who do and do not exhibit oral-stool microbial community coalescence. Plotted are the top 10 families by P-value using Mann-Whitney test with FDR-adjustment. *P < 0.01, **P < 0.001.

We next sought to identify baseline microbiome features predictive of developing coalescence. Stool Shannon Diversity (P=0.007) and the number of observed OTUs (P=0.031) were significantly lower at baseline in patients who subsequently exhibited oral-stool community coalescence (**Figure 6A**). However, there was no statistically significant difference in baseline α-diversity of among oral samples between those who did or did not develop coalescence. There were a number of taxonomic differences in both the oral and the stool for those who coalesced versus those who did not. Using LEfSe, we identified the enrichment of organisms such as *Lactococcus*, *Lactobacillus* and *Xanthamonas* among baseline stool samples from patients that exhibited microbial community coalescence compared to patients that did not. There was also enrichment of taxa such as *Enterobacter*, *Lactococcus*, and *Pseudomonadales* among baseline oral samples from patients that exhibited microbial community coalescence compared to patients that did not (**Figure S5**). By the time of neutrophil recovery (end of study), the relative abundance of Bacteroidia (P<0.001), Fusobacteria (P=0.012), and Clostridia (P=0.013) in the stool were significantly lower in patients with oral-gut community coalescence (**Figure 6B**).

**Figure 7:**
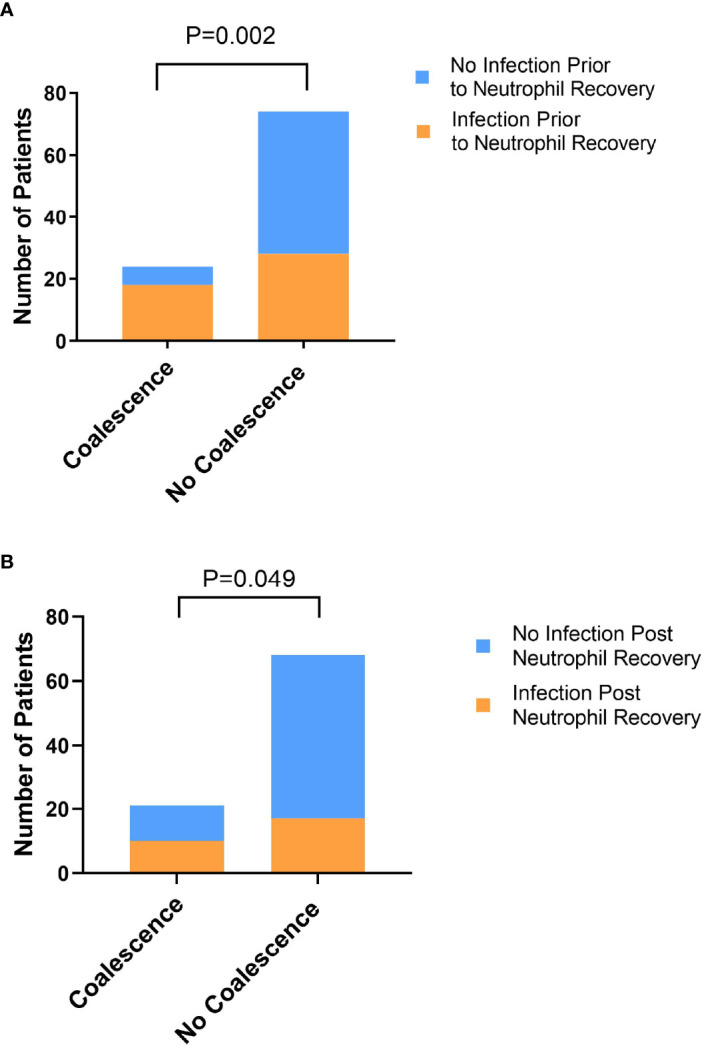
Exhibiting oral-stool community coalescence was associated with the occurrence of infections prior to neutrophil recovery and in the 90 days post neutrophil recovery. Pearson’s χ2 test was used to determine whether there was a significant difference between the frequency of infection prior to neutrophil recovery **(A)** and in the 90 days post-neutrophil recovery **(B)** between those who did and did not exhibit oral-stool coalescence during induction chemotherapy.

Inasmuch as previous studies have identified lower baseline stool α-diversity and domination events as being associated with infectious risk in patients with hematologic malignancy (Olsen and Yamazaki, 2019), we assessed whether coalescence was associated with infectious outcomes prior to neutrophil recovery and in the 90 days post neutrophil recovery. Indeed, the association between coalescence and infections prior to neutrophil recovery (χ2 = 10.05, P=0.002) (**Figure 7**), as well as infections post neutrophil recovery (χ2 = 3.88, P=0.049), was statistically significant using a χ2 test. The odds of both an infection prior to neutrophil recovery (OR=4.93, 95% CI 1.83-13.92) and an infection in the 90 days post neutrophil recovery (OR=2.72, 95% CI 1.03-7.1) were higher if a patient developed oral and stool microbial community coalescence during induction chemotherapy.

### Antibiotic Exposure Drives Oral and Stool Microbial Community Coalescence

Given that patients who exhibited coalesced communities demonstrated specific stool taxonomic decreases and that coalescence tended to occur some three weeks into leukemia therapy, we hypothesized that antimicrobial use might be a major contributor to these changes, and consequently, oral-stool community coalescence. Thus, we sought to understand if particular antibiotic exposures were associated with oral-stool microbiome coalescence in our cohort. The use of antibiotics as either prophylaxis against or empiric treatment for neutropenic fever is presented in **Supplementary Table 2** and summarized in **Figure S6**. Using a time-varying Cox proportional hazards model to analyze cumulative antibiotic exposure (**Table 1**), each additional day of cefepime (HR 1.10, 95% CI 1.01 – 1.18, P = 0.021), linezolid (HR 1.15, 95% CI 1.06 – 1.24, P <0.001), meropenem (HR 1.13, 95% CI 1.05 – 1.21, P = 0.001) and metronidazole (HR 1.13, 95% CI 1.05 – 1.21, P = 0.001) use increased the hazard of oral-stool microbial coalescence. On the other hand, levofloxacin (HR 0.75, 95% CI 0.61 – 0.93, *P* = 0.009) appeared to have protective effects against microbiome community coalescence.

**Table 1:** Time-varying analysis of antibiotic exposure during the risk period for patients who do and do not exhibit oral-stool microbial coalescence.

Antibiotic	Cumulative antimicrobial exposure			Any antimicrobial exposure		
	HR^a^	95% CI^a^	*P* ^a^	HR^b^	95% CI^b^	*P* ^b^
**Amikacin**	1.08	0.83-1.40	0.555	1.69	0.61-4.65	0.310
**Cefepime**	1.10	1.01-1.18	0.021	2.83	1.16-6.90	0.022
**Cefpodoxime**	1.00	0.89-1.12	0.949	0.93	0.40-2.19	0.866
**Ciprofloxacin**	1.06	0.98-1.15	0.138	1.83	0.79-4.23	0.159
**Daptomycin**	1.06	0.95-1.18	0.294	1.35	0.55-3.34	0.510
**Ertapenem**	1.05	0.92-1.20	0.447	1.12	0.43-2.92	0.812
**Levofloxacin**	0.75	0.61-0.93	0.009	0.15	0.05-0.42	<0.001
**Linezolid**	1.15	1.06-1.24	<0.001	4.60	1.14-18.61	0.032
**Meropenem**	1.13	1.05-1.22	0.001	6.72	2.09-21.76	0.001
**Metronidazole**	1.13	1.05-1.21	0.001	6.03	2.48-14.65	<0.001
**Piperacillin-** **Tazobactam**	1.01	0.93-1.10	0.836	0.96	0.37-2.45	0.927
**Tigecycline**	1.08	0.98-1.20	0.134	2.61	1.06-6.42	0.036

a Hazard ratios (HR), confidence intervals (CI), and P-values refer to hazard associated with each additional day of antibiotic exposure (cumulative exposure).

b Hazard ratios (HR), confidence intervals (CI) and P-values refer to hazard associated with any antibiotic exposure once exposed.

Analyzing *any* antibiotic exposure as a time-varying covariate (**Table 1**) produced analogous results for cefepime (HR 2.81, 95% CI 1.16 – 6.90, *P* = 0.022), linezolid (HR 4.60, 95% CI 1.14 – 18.61, *P* = 0.032), meropenem (HR 6.72, 95% CI 2.08 – 21.76, *P* = 0.001), and metronidazole (HR 6.03, 95% CI 2.48-14.65, *P <*0.001). Once exposed, all aforementioned antibiotics were associated with an increased risk of coalescence except for levofloxacin (HR 0.15, 95% CI 0.05 – 0.42, *P*<0.001), which appeared to be associated with a decreased risk. Analyzing any antibiotic exposure as a time-varying covariate additionally added tigecycline (HR 2.61, 95% CI 1.06 – 6.42, *P* = 0.036) as a factor associated with an increased risk of coalescence.

## Discussion

While it has been demonstrated that healthy intra-individual gut and oral microbiomes typically have distinct compositions (Human Microbiome Project, 2012; Rashidi et al., 2021), it has recently been shown that oral bacteria can colonize the gut even in healthy individuals but particularly so in diseased states (Qin et al., 2014; Dickson et al., 2016; Atarashi et al., 2017; Schmidt et al., 2019). The term “microbial community coalescence” is used to describe distinct community interchange events among different environments, including those between body habitats (Rillig et al., 2015). In this manuscript, we have identified taxonomic overlap in ~25% of oral and stool samples from patients undergoing acute leukemia therapy (**Figures 1**, **2**), indicating the presence of significant community coalescence in this acutely ill cohort. Moreover, this coalescence was strongly associated with specific antibiotic exposures, such as metronidazole and meropenem (**Tables 1**, **S2**), and appeared to have implications for infectious complications both during and after chemotherapy receipt (**Figure 7**).

Coalescence in acute myeloid leukemia patients undergoing induction chemotherapy was characterized by increasingly shared OTUs compared to unique OTUs between intra-patient oral and stool pairs over time (**Figure 3**). Additionally, oral sample α-diversity had a steeper decrease over time in patients who experienced coalescence compared to those that did not (**Figure 4**). While stool samples from coalesced patients had a similar trajectory (slope or change in diversity) over time compared to non-coalesced patients, those who developed oral-stool community coalescence prior to neutrophil recovery from induction chemotherapy had an overall lower baseline α-diversity to start (**Figures 4**, **6**). Interestingly, although we found oral-associated taxa (such as *Veillonella*, *Oribacterium*, and *Actinomyces*) in the stool across all AML patients (**Figure S4**), there was a similar abundance of *Streptococcus* and *Stenotrophomonas* in both oral and stool samples of coalesced patients, which was not present in non-coalesced patients, where *Streptococcus* and *Stenotrophomonas* were significantly higher in the oral cavity (**Figure 5**).

A key finding of our study was that specific antimicrobials, namely meropenem, cefepime, and metronidazole, were associated with coalescence (**Tables 1**, **S2**). The findings for meropenem and metronidazole have a logical mechanistic basis given that both agents have strong anti-anaerobic activity and are generally used as second-line agents in leukemia patients at our institution. Patients receiving such agents have typically received a high load of antimicrobials and lost anaerobic GI flora, likely predisposing the distal GI tract to invasion by aero-tolerant oral flora. Indeed, we have previously shown in this cohort that receipt of carbapenems was associated with loss of stool microbial diversity (Galloway-Pena et al., 2016; Galloway-Pena et al., 2017; Galloway-Pena et al., 2020). Moreover, in this analysis we found that coalescence was associated with lower abundances of key anaerobic components of the intestinal tract such as Bacteroides, Clostridia, and Fusobacteria by the time of neutrophil recovery (**Figure 6B**). In contrast, the association of cefepime with coalescence was somewhat unexpected given that it is used as a front-line agent and is not thought to have marked impact on anaerobes. The cefepime association was particularly surprising given that receipt of piperacillin-tazobactam, another first line agent but one that has a broader anti-anaerobic spectrum than cefepime, was not associated with coalescence. Interestingly, it has been reported that receipt of cefepime relative to piperacillin-tazobactam was more strongly associated with infection of drug-resistant organisms capable of colonizing the oropharynx of hospitalized patients such as methicillin-resistant *Staphylococcus aureus* and *Pseudomonas aeruginosa* (Ginn et al., 2012). Thus, our data suggest that development of coalescence is likely more complex than simply reflecting the loss of anaerobic organisms from the GI tract.

Another key finding of our study was that receipt of levofloxacin but not cefpodoxime was negatively associated with coalescence development (**Tables 1**, **S2**). Both agents are used in the prophylactic setting in our institution with levofloxacin being the first choice and cefpodoxime reserved for patients who have some contraindication to fluoroquinolone use (Doan et al., 2019). One relatively simple explanation for the negative association of levofloxacin and coalescence would be that patients who stay free from neutropenic fever, and thus are not given broad-spectrum antimicrobials, would remain on levofloxacin throughout the study. In this scenario, the negative association between levofloxacin and coalescence should be particularly strong when considering cumulative levofloxacin receipt. However, we observed that the negative association was strongest for *any* levofloxacin exposure. In concert with our data, a recent study from the University of Pennsylvania found that levofloxacin exposure during treatment of hematologic malignancy was associated with increased microbial diversity and no loss of key anaerobic taxa (Ziegler et al., 2019). The lack of a negative association between cefpodoxime and coalescence also argues against levofloxacin receipt simply being a marker for avoidance of treatment with other antimicrobials inasmuch as cefpodoxime and levofloxacin prophylaxis appear to be associated with similar rates of neutropenic fever and antimicrobial exposure in hematologic malignancy patients (Doan et al., 2019). Herein, there was no statistically significant difference in total days on antibiotics (Wilcoxon rank test P=0.07) or the number of different antimicrobials received (Student t-test P=0.40) between patients who received levofloxacin and those that receive cefopodoxime. We speculate that cefpodoxime may be affecting the microbiome in a similar manner to that observed for cefepime, given the similarities between the two drugs.

Similar to the patterns observed in our cohort, dominance of pathogens at multiple sites and loss of site specificity was previously observed in critically ill children (Rogers et al., 2016). Disruption of the microbial community associated with individual operational taxonomic units (OTUs) dominating a community at multiple body sites has been reported in adult ICU patients as well (Zaborin et al., 2014; McDonald et al., 2016). These data incite the necessity for understanding the causal relationships between the altered microbiome at multiple sites, infections, and systemic diseases (Olsen and Yamazaki, 2019; Park et al., 2021). However, although a high percentage of coalesced patients experienced a domination event by the same taxa in both the oral and stool, these events were mostly transient. Because the overall number of shared OTUs between the oral and stool samples increase over time, this suggests this phenomenon is not just a reflection of domination by one bacteria at two sites, but possible oralization of the gut (Horvath et al., 2019; Olsen and Yamazaki, 2019; Park et al., 2021; Horvath et al., 2021). Therefore, the mechanism for coalescence and consequent infection is likely that lower baseline stool microbiome diversity in combination with gut decontamination with specific antibiotic administration creates a permissive environment for coalescence to occur. Loss of site specificity and dominance by these organisms at both sites, as seen by the presence of similar communities in both the oral cavity and the stool, was associated with infectious complications in our leukemia patients. Future directions include understanding which physiological features (e.g., pH or mucin integrity in the gut) are altered as a consequence of prolonged antibiotic exposure and/or chemotherapy which may lead to loss of site-specific communities (Park et al., 2021).

Our study extends previous understanding of dysbiosis in hospitalized and critically ill patients. However, there are shortcomings of our study worth noting. Given that we used 16S rRNA data, we could not track populations at the resolution necessary to establish directionality at the strain level. Therefore, although we can infer community and taxa level dynamics, we cannot be certain of specific species or strain transmission between the oral cavity and the gut. Moreover, it is unclear how the hospital context, colonization of hospital surfaces, and potential of transmission might convolute analysis of cross-microenvironment transfer. Furthermore, increasing the size of the cohort by extending these analyses to multiple institutions would better power these studies and account for institution-specific antimicrobial administration differences (Anderson et al., 2011; Maurer et al., 2013). Additionally, given this study is observational, establishing the influence of different antibiotic and antimicrobial regimens on coalescence would require a prospective randomized study.

In summary, the data herein shed new light on the emerging concept and clinical consequences of oral-stool microbiome coalescence in hospitalized patients. Increased understanding of the host, microbiome, and antimicrobial factors driving such coalescence could assist with novel preventive or therapeutic strategies.

## Data Availability Statement

Publicly available datasets were analyzed in this study. This data can be found here: the NCBI Sequence Read Archive (http://www.ncbi.nlm.nih.gov/sra) under the BioProject IDs PRJNA352060 and PRJNA526551.

## Ethics Statement

The studies involving human participants were reviewed and approved by the MD Anderson Cancer Center Institutional Review Board (PA13‐0339) and conducted in compliance with the Declaration of Helsinki. The patients/participants provided their written informed consent to participate in this study.

## Author Contributions

JG-P, CP, and SS were responsible experimental study design. PS, ND, and DK were responsible for clinical study design, collection of samples, and clinical data. SF, SA, YS, SR, and JG-P were responsible for experimentation and data analysis. SF and JG-P wrote the manuscript. All authors edited and reviewed manuscript. All authors contributed to the article and approved the submitted version.

## Funding

This work was supported by the MD Anderson Cancer Center “Knowledge Gap” and “Multidisciplinary Research Program” funding mechanisms to DK and SS. JG-P was supported by the MD Anderson Odyssey Fellowship Program and the CFP Foundation for part of the duration of this work as well as the NIH (1 K01 AI143881-01 from the NIAID). SR is supported by the NSF Graduate Research Fellowship DGE 1842494 and, for a portion of this work, was supported by the NIH T32 Grant CA96520-13: Training program in Biostatistics for Cancer Research. CP is partially supported by NIH/NCI Cancer Center Support Grant P30CA016672 (Biostatistics Resource Group). DK is supported by the Texas 4000 Distinguished Professorship for Cancer Research. DK acknowledges the Robert C. Hickey Chair for Clinical Care endowment (to DK). SF is partially supported by the TAMU Association of Former Students Graduate Merit Fellowship and the Interdisciplinary Degree Program of Genetics.

## Conflict of Interest

SA has received research support from Melinta Therapeutics and has served on advisory boards for Shionogi, Paratek, and Merck. DK reports honoraria and research support from Gilead Sciences, received consultant fees from Astellas Pharma, Merck, and Gilead Sciences, and is a member of the Data Review Committee of Cidara Therapeutics, AbbVie, Synegiis and the Mycoses Study Group.

The remaining authors declare that the research was conducted in the absence of any commercial or financial relationships that could be construed as a potential conflict of interest.

## Publisher’s Note

All claims expressed in this article are solely those of the authors and do not necessarily represent those of their affiliated organizations, or those of the publisher, the editors and the reviewers. Any product that may be evaluated in this article, or claim that may be made by its manufacturer, is not guaranteed or endorsed by the publisher.
