# Supplementary material for: Oral and Stool Microbiome Coalescence and Its Association With Antibiotic Exposure in Acute Leukemia Patients
Source: Front Cell Infect Microbiol. 2022 Mar 31;12:848580. doi: 10.3389/fcimb.2022.848580 (PMC9010033; doi:10.3389/fcimb.2022.848580)
Supplement: Supplementary file 1 [file DataSheet_1.pdf]

Supplemental Table 1. Patients Who Coalescence also Exhibit Domination Events in Both Oral and Stool Samples by Distinct Genera

**Genus**	**Percent of Coalescence Patients Experiencing Domination Events at Both Oral and Stool Sites (n)**
Streptococcus	**52.1% (12)**
Staphylococcus	**17.4% (4)**
Enterococcus	**13.0% (3)**
Lactobacillus	**13.0% (3)**
Stenotrophomonas	**13.0% (3)**

Supplemental Table 2. Bivariate comparison of antibiotic exposure during the risk period for patients who do and do not exhibit oral-stool microbial coalescence.

Antibiotic	Coalescence (n=23)	No Coalescence (n=70)	p-value[a]
**Amikacin**			
Median Number of Days (IQR)	0.00 (0-0)	0.00 (0-0)	0.745
Any use (n, %)	5 (22)	13 (19)	0.765
**Cefepime**			
Median Number of Days (IQR)	4.00 (0-9)	2.00 (0-6)	0.120
Any use (n, %)	17 (74)	38 (54)	0.142
**Cefopodoxime**			
Median Number of Days (IQR)	0.00 (0-3)	0.00 (0-2)	0.720
Any use (n, %)	7 (30)	20 (29)	1.000
**Ciprofloxacin**			
Median Number of Days (IQR)	0.00 (0-5)	0.00 (0-1)	0.273
Any use (n, %)	9 (39)	19 (27)	0.303
**Daptomycin**			
Median Number of Days (IQR)	0.00 (0-1)	0.00 (0-0)	0.598
Any use (n, %)	6 (26)	15 (21)	0.774
**Ertapenem**			
Median Number of Days (IQR)	0.00 (0-0)	0.00 (0-0)	0.995
Any use (n, %)	5 (22)	17 (24)	1.000
**Levofloxacin***			
Median Number of Days (IQR)	0.00 (0-2)	6.00 (0-13)	<0.001
Any use (n, %)	6 (26)	52 (74)	<0.001
**Linezolid***			
Median Number of Days (IQR)	8.00 (4-11)	5.00 (0-8)	0.019
Any use (n, %)	21 (91)	52 (74)	0.141
**Meropenem***			
Median Number of Days (IQR)	5.00 (1-17)	0.00 (0-6)	0.007
Any use (n, %)	19 (83)	34 (49)	0.007
**Metronidazole***			
Median Number of Days (IQR)	2.00 (0-4)	0.00 (0-0)	<0.001
Any use (n, %)	14 (61)	9 (13)	<0.001
**Piperacillin-Tazobactam**			
Median Number of Days (IQR)	0.00 (0-4)	0.00 (0-0)	0.959
Any use (n, %)	6 (26)	19 (27)	1.000
**Tigecycline**			
Median Number of Days (IQR)	0.00 (0-4)	0.00 (0-0)	0.047
Any use (n, %)	9 (39)	12 (17)	0.043

[a] When comparing cumulative antibiotic exposure between those do and do not (reference) exhibit coalescence p-values are from Wilcoxon rank-sum test, when comparing any exposure Fisher's exact test was used.
