# Supplementary material for: Oral and Stool Microbiome Coalescence and Its Association With Antibiotic Exposure in Acute Leukemia Patients
Source: Front Cell Infect Microbiol. 2022 Mar 31;12:848580. doi: 10.3389/fcimb.2022.848580 (PMC9010033; doi:10.3389/fcimb.2022.848580)
Supplement: Supplementary file 2 [file DataSheet_2.pdf]

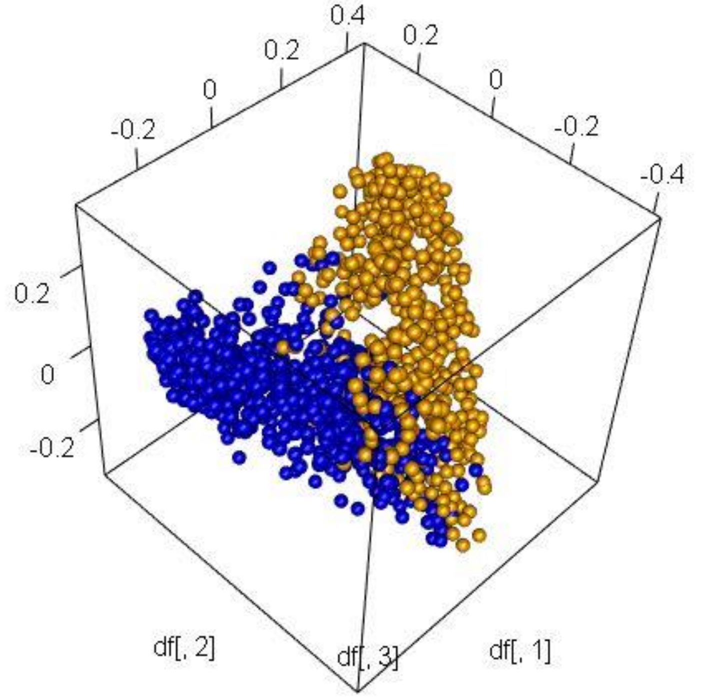

Figure S1. 3D PCoA plot of unweighted UniFrac distances of oral and stool samples. A 3-dimensional principal coordinates analysis plot of the unweighted Unifrac distances of the 737 oral samples and 551 fecal samples using the package plot3Drgl in R. Oral (blue) and stool (orange) samples are colored by sample site.

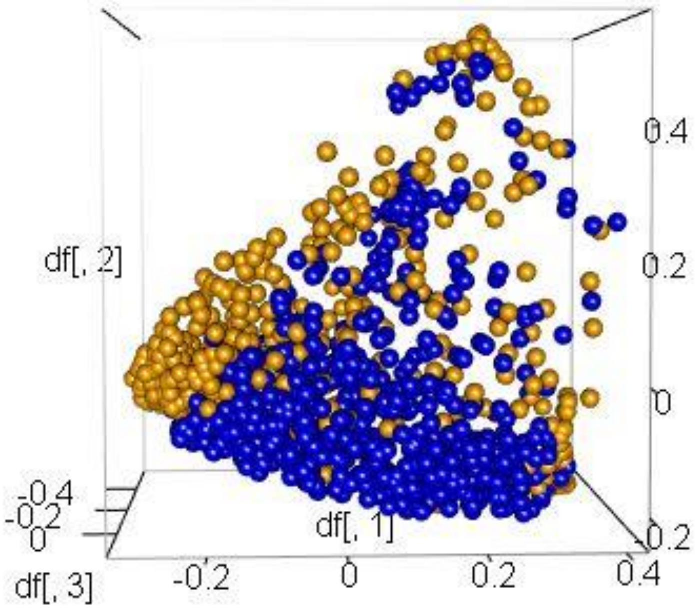

Figure S2. 3D PCoA plot of weighted UniFrac distances of oral and stool samples. A 3-dimensional principal coordinates analysis plot of the weighted Unifrac distances of the 737 oral samples and 551 fecal samples using the package plot3Drgl in R. Oral (blue) and stool (orange) samples are colored by sample site.

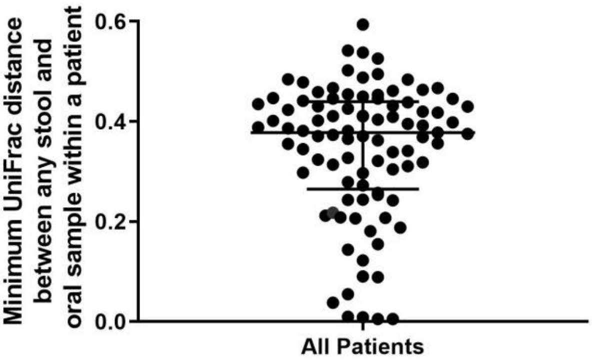

Figure S3. Oral and stool community coalescence is defined by using intra-patient minimum UniFrac distances between oral-stool pairs. Each data point represents the minimum UniFrac distance (from the matrix) between any oral-stool pair from the longitudinal sampling within an individual patient. Plotted is the median and interquartile range. Those that fell below the interquartile range were defined as exhibiting oral-stool microbial community coalescence.

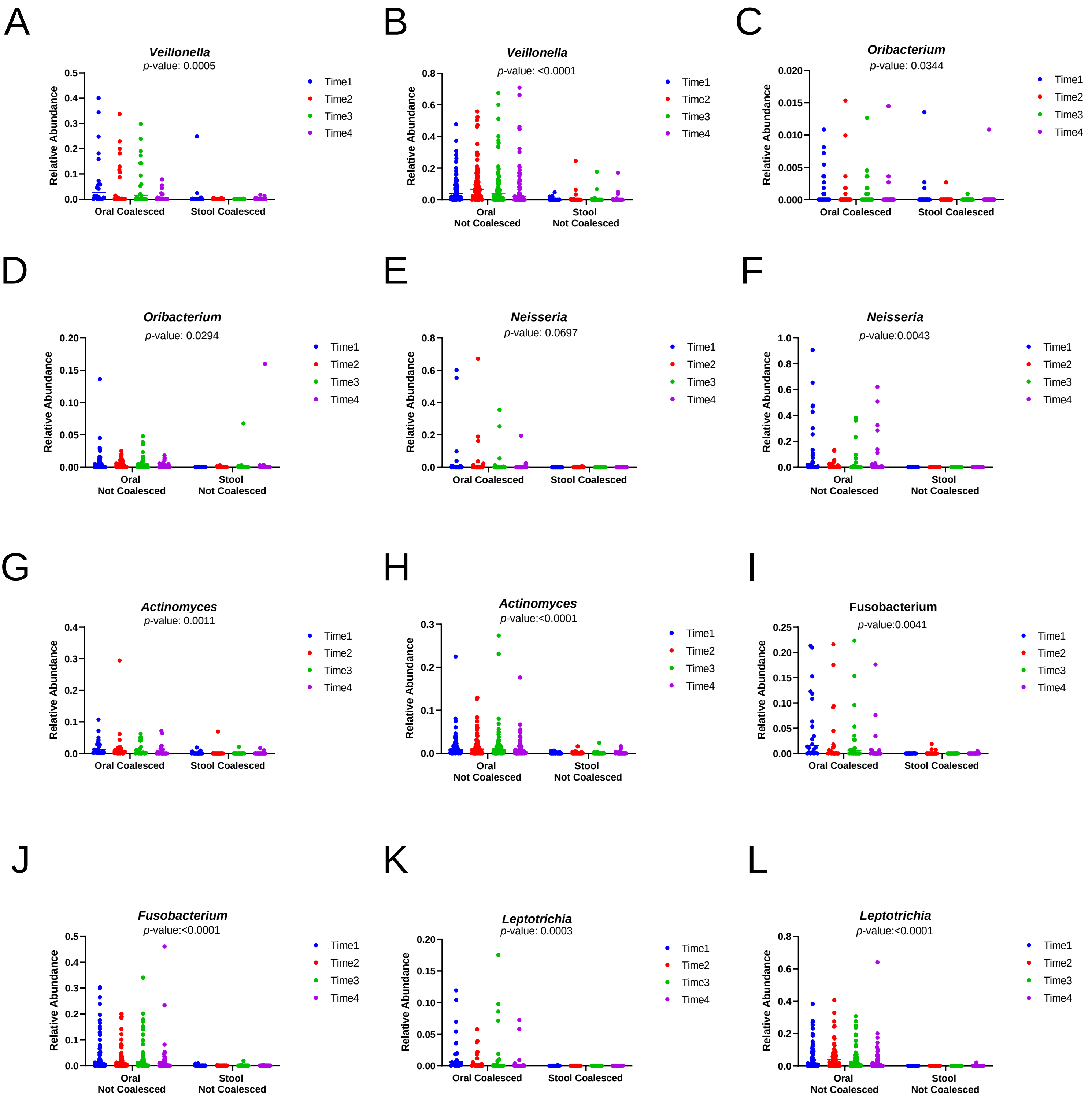

Figure S4. The relative abundance of oral associated bacteria in oral and stool samples of AML patients. The relative abundance of selected oral bacteria (A-B) *Veillonella*, (C-D) *Oribacterium*, (E-F) *Neisseria*, (G-H) *Actinomyces*, (I-J) *Leptotrichia*, and (K-L) *Fusobacterium* was plotted for oral and stool samples. The color scale indicates sampling time point for each of the patient samples, where blue is the first timepoint sampled, and purple is the last sampling time point. P values were calculated between oral and stool samples utilizing a mixed model for repeated measures.

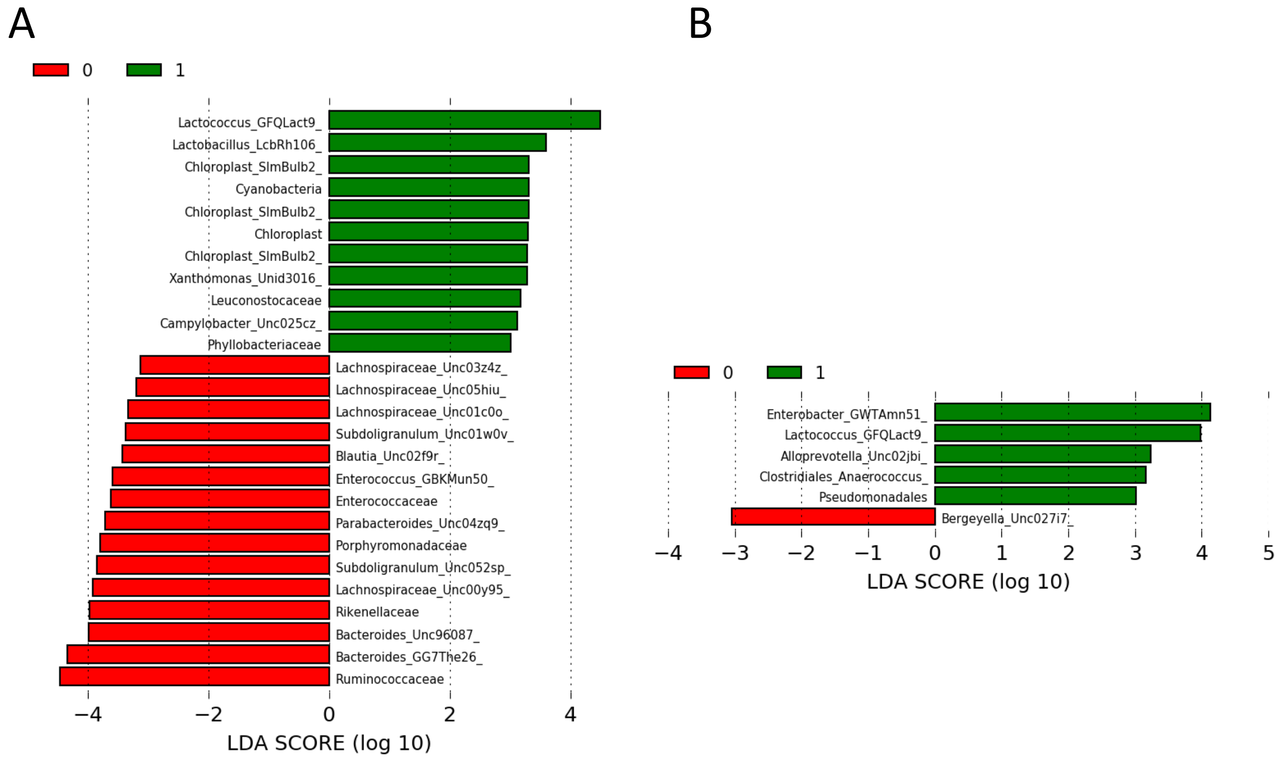

Figure S5. Differential baseline taxonomic abundances among patients who coalesed and did not coalese. Linear discriminant analysis of effect size (LEfSe) of taxonomic abundances for baseline samples of coalesced (1) and not coalesced (0) patients. Horizontal bars represent the effect size for each taxon, the length of each bar represents the log10 transformed LDA score, indicated by the vertical dotted lines. The grouped data was analyzed using the Kruskal-Wallis test with a significant set to 0.05 to determine differentially abundant taxa. Differentially abundant taxa that are present in patients that experienced coalescence are indicated by green, and taxa that are present in patients who did not experience coalescence appear in red. The threshold for the logarithmic LDA score for discriminant features was set to 2.0. A) is stool samples. B) is oral samples.

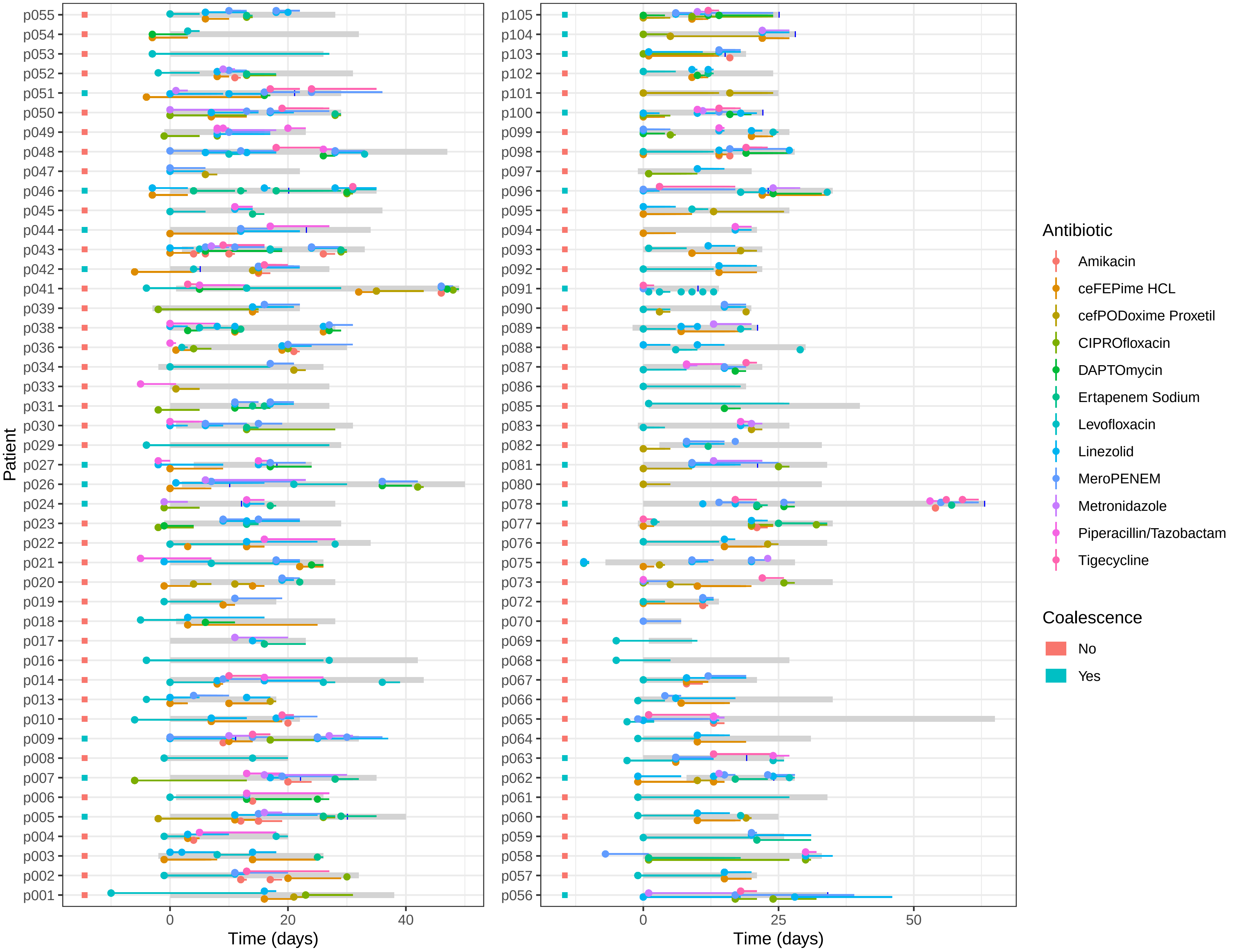

Patient
Time (days)
Antibiotic
Amikacin
ceFEPime HCL
cefPODoxime Proxetil
CIPROfloxacin
DAPTOmycin
Ertapenem Sodium
Levofloxacin
Linezolid
MeroPENEM
Metronidazole
Piperacillin/Tazobactam
Tigecycline
Coalescence
No
Yes
p055 p054 p053 p052 p051 p050 p049 p048 p047 p046 p045 p044 p043 p042 p041 p039 p038 p036 p034 p033 p031 p030 p029 p027 p026 p024 p023 p022 p021 p020 p019 p018 p017 p016 p014 p013 p010 p009 p008 p007 p006 p005 p004 p003 p002 p001
p105 p104 p103 p102 p101 p100 p099 p098 p097 p096 p095 p094 p093 p092 p091 p090 p089 p088 p087 p086 p085 p083 p082 p081 p080 p078 p077 p076 p075 p073 p072 p070 p069 p068 p067 p066 p065 p064 p063 p062 p061 p060 p059 p058 p057 p056
0 20 40
0 25 50

Figure S6. Summary plot of patient antibiotic administration, sampling period, and coalescence determination. The x-axis shows the duration of sampling and antibiotic administration. Colored dots and lines represent the first administration and continuation of antibiotic treatment. The grey boxes show the sampling period for each patient. The coalescence designation is represented by pink and blue boxes. If coalescence was observed, the time of coalescence is represented within the grey box by a blue band.
